# Strategies for the Preparation of Phosphorus Janus Dendrimers and Their Properties

**DOI:** 10.3390/molecules28145570

**Published:** 2023-07-21

**Authors:** Joel Cejas-Sánchez, Anna Kajetanowicz, Karol Grela, Anne-Marie Caminade, Rosa María Sebastián

**Affiliations:** 1 Biological and Chemical Research Centre, Faculty of Chemistry, University of Warsaw, Żwirki i Wigury 101, 02-089 Warsaw, Poland; j.cejassanch@student.uw.edu.pl (J.C.-S.); a.kajetanowicz@uw.edu.pl (A.K.); klgre@uw.edu.pl (K.G.); 2 Department of Chemistry, Universitat Autònoma de Barcelona, Cerdanyola del Vallès, Bellaterra, 08193 Barcelona, Spain; 3 Centro de Innovación en Química Avanzada (ORFEO-CINQA), Universitat Autònoma de Barcelona, Cerdanyola del Vallès, Bellaterra, 08193 Barcelona, Spain; 4 Laboratoire de Chimie de Coordination du CNRS, 205 Route de Narbonne, BP 44099, CEDEX 4, 31077 Toulouse, France; 5 Université de Toulouse, UPS, INPT, CEDEX 4, 31077 Toulouse, France

**Keywords:** phosphorus chemistry, Janus dendrimers, dendrons, ^31^P NMR spectroscopy

## Abstract

Dendrimers, being highly branched monodispersed macromolecules, predominantly exhibit identical terminal functionalities within their structural framework. Nonetheless, there are instances where the presence of two distinct surface functionalities becomes advantageous for the fulfilment of specific properties. To achieve this objective, one approach involves implementing Janus dendrimers, consisting of two dendrimeric wedges terminated by dissimilar functionalities. The prevalent method for creating these structures involves the synthesis of dendrons that possess a core functionality that complements that of a second dendron, facilitating their coupling to generate the desired dendrimers. In this comprehensive review, various techniques employed in the fabrication of phosphorus-based Janus dendrimers are elucidated, displaying the different coupling methodologies employed between the two units. The advantages of phosphorus dendrimers over classic dendrimers will be shown, as the presence of at least one phosphorus atom in each generation allows for the easy monitoring of reactions and the confirmation of purity through a simple technique such as ^31^P NMR, as these structures typically exhibit easily interpretable patterns.

## 1. Introduction

Dendrimers are highly branched macromolecules characterized in most cases by a spherical shape. This unique class of polymers is often constructed through step-by-step reactions, starting from a polyfunctional core and progressively growing over generations. At each generation, new branching points are introduced, leading to an exponential increase in the number of terminal functional groups [1,2]. Dendrimers have garnered significant attention in the scientific literature and are considered versatile systems with a wide range of applications in various fields such as biology [3], biomedicine [1,4], material science [5,6], catalysis [7,8,9], or sensors [10,11], among others. However, the functionalization of dendrimer surfaces has been predominantly limited to a uniformity of functional groups, restricting their applicability to a single specific area.

To overcome this limitation and enable the incorporation of multiple types of terminal groups on dendrimer surfaces, several methodologies have been developed. These approaches allow for the introduction of various functional groups, enabling the synthesis of dendrimers with combined properties within a single molecule. One common method involves grafting two types of functions on the surface, either in a stochastic or precise manner, by sequentially reacting one functionality atop another in a stepwise synthesis [12]. However, the most intriguing approach involves the preparation of dendrimers with a multifunctional surface using dendrimeric structures composed of two dendrons (wedge-shaped dendrimeric units) through a central core coupling [13,14]. These structures are commonly referred to as Janus dendrimers [15], named after the Roman god of transitions and beginnings, depicted with two faces oriented in opposite directions. It is worth noting that Janus dendrimers have also been referred to by different names in the literature, such as surface block dendrimers [16] or di-block dendrimers [17].

**Scheme 1:**
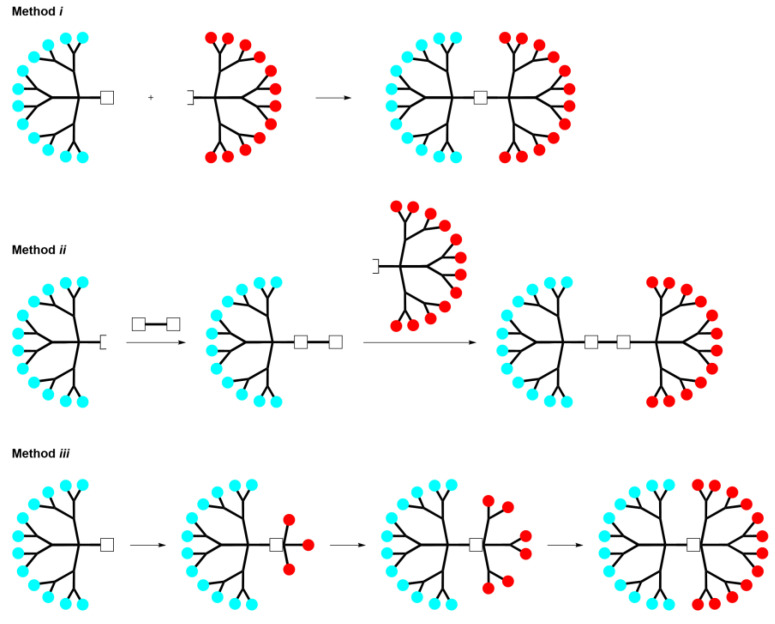
Schematic representation of the main methods to synthesize Janus dendrimers with two functionalities at the surface.

Three main methodologies have been developed for the synthesis of Janus dendrimers, as depicted in Scheme 1. These methods involve either the reaction of two dendrons with complementary functions at the core level (method *i*); the sequential reaction of a first dendron with a well-defined core, followed by grafting a second dendron onto the remaining core functions (method *ii*); or the divergent growth of new branches from a focal point of a dendron (method *iii*).

**Scheme 2:**
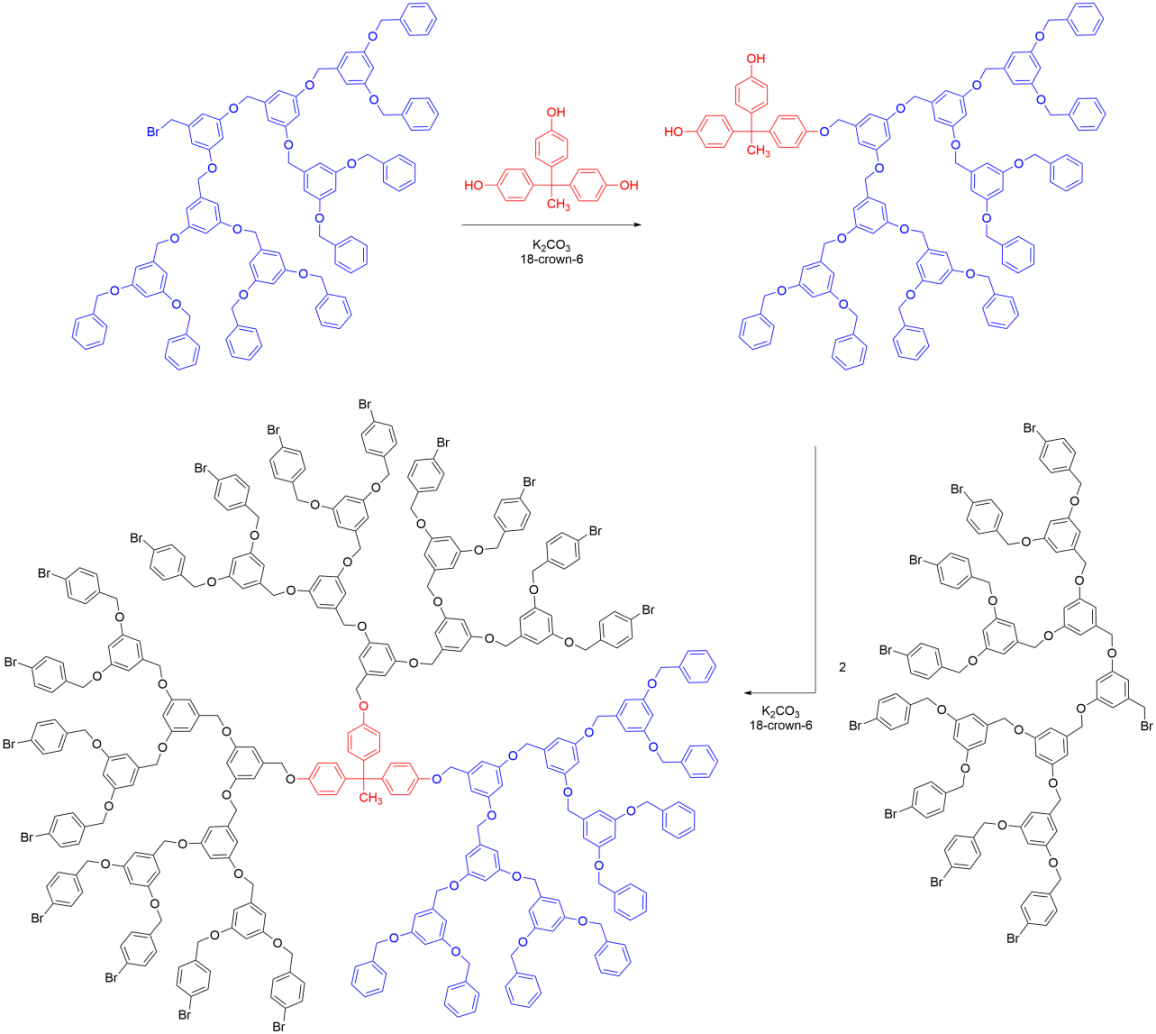
Synthetic strategy for the preparation of a PBzE Janus dendrimer with a polyphenol core, based on method *ii*.

Janus dendrimers have been the subject of interest since their first description by Fréchet et al. in 1991 [18]. In their pioneering work, a Janus dendrimer was synthesized through a convergent approach by coupling two poly(benzyl ether) (PBzE) dendrons. One dendron, owning a benzyl bromide group, was grafted onto a polyphenolic core through a monosubstitution reaction under basic conditions, resulting in an AB_2_-type core (one function different from the two others). The remaining unreacted phenol groups on the core were then reacted with other dendrons possessing different functionalities to complete the synthesis of the Janus dendrimer (Scheme 2).

The synthesis of PBzE Janus dendrimers via substitution reactions involving phenols and benzylic halides has been widely employed in the literature. This approach has enabled the creation of Janus dendrimers with benzyl/carboxylate terminal groups or benzyl/nitrile functionalities [19]. These PBzE Janus dendrimers have found diverse applications, such as the formation of liquid membranes for organic/water interphase systems and the modification of polymer properties in terms of dipole moment [20] or glass transition temperatures [21], among others.

**Figure 1:**
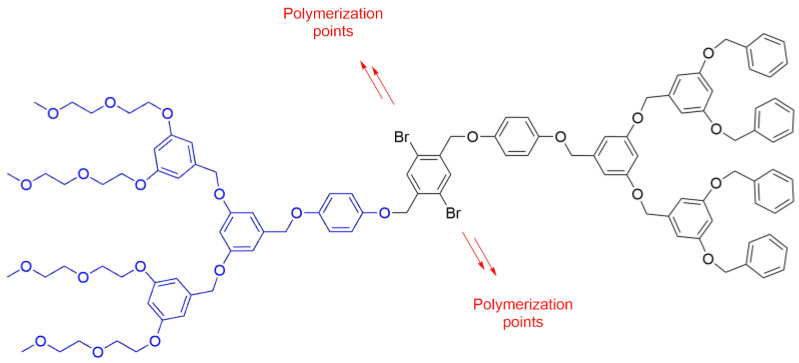
PBzE Janus dendrimer holding OEG chains on one side and bromine atoms at the core to be used as polymerization points, synthesized by method *i*.

Interestingly, a polymerizable PBzE Janus dendrimer was also synthesized using a similar method, wherein a dendron terminated with oligoethylene (OEG) chains was reacted with a second dendron ended with benzyl groups and possessing a dibromobenzene unit at core (Figure 1). Later, this dibromobenzene group was reacted with diboronic acid in a polycondensation reaction to afford the desired polymeric structure [22].

**Scheme 3:**
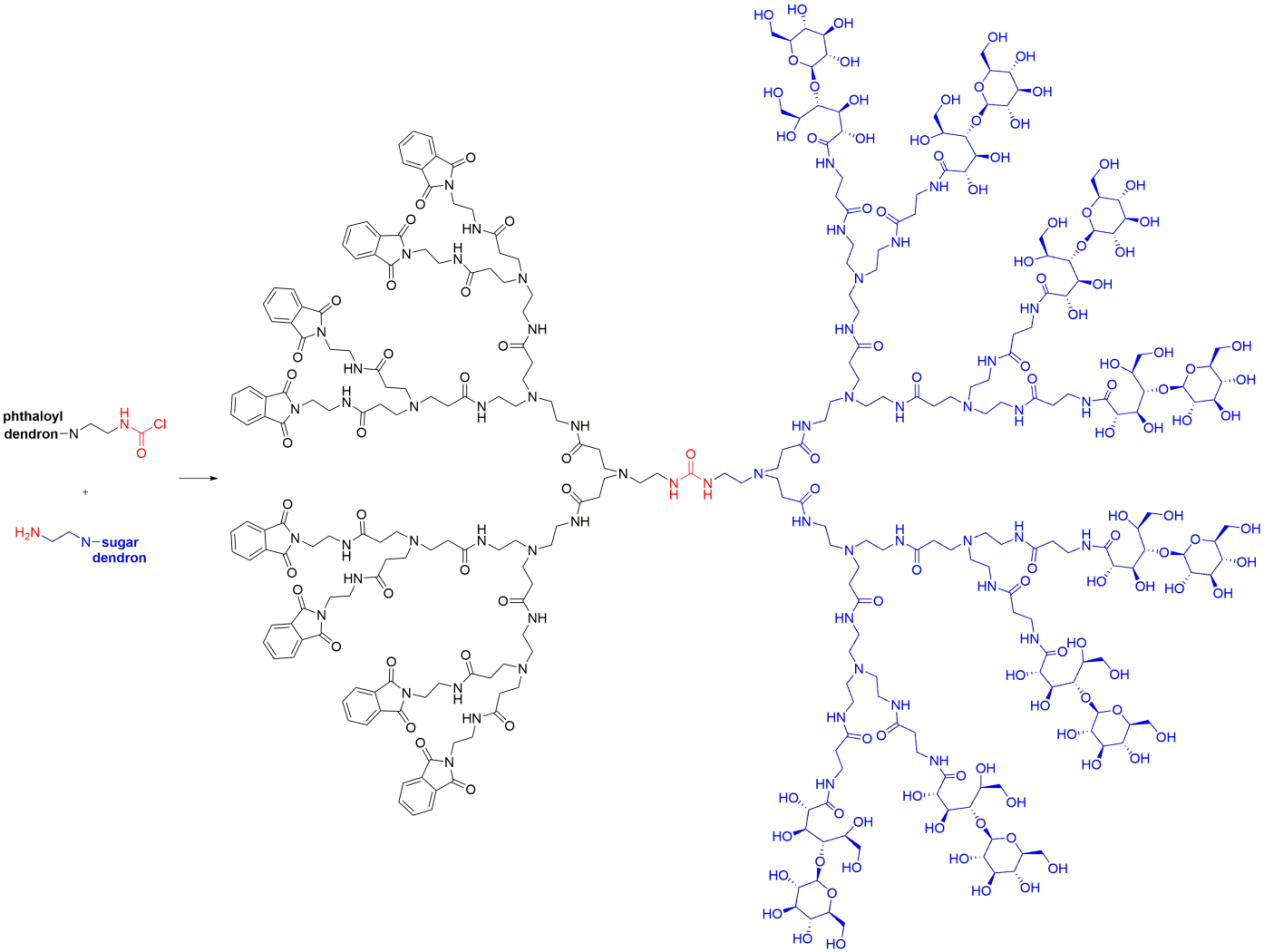
PAMAM Janus dendrimer bearing phthaloyl groups on one side and sugars on the other, prepared by method *i*.

In addition to PBzE dendrimers, classic poly(amidoamine) (PAMAM) structures have also been used for the synthesis of Janus dendrimers. For instance, Aoi et al. [23] demonstrated the synthesis of a Janus dendrimer by grafting a dendron bearing a primary amine core and terminated with sugar chains onto a second PAMAM dendron possessing phthaloyl groups on the surface through the formation of a urea linker by a reaction between a carbamoyl chloride and a primary amine (Scheme 3). They can be potentially used in the fields of supramolecular self-assembly or biology and biomedicine by means of binding surface sectors to proteins and DNA.

**Scheme 4:**
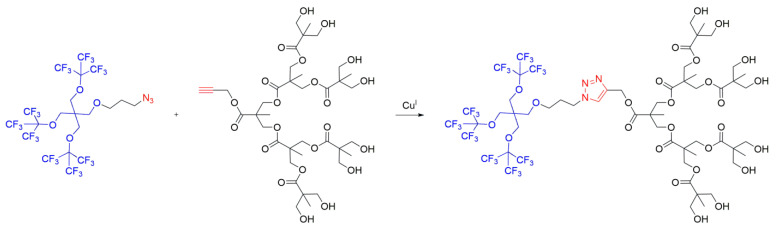
Fluorinated Janus dendrimer synthesized by “click” reaction of two dendrons containing azide and acetylene moieties, based on method *i*.

An intriguing Janus dendrimer was recently designed by Rosati et al. [24], employing a novel linker using “click” chemistry (Scheme 4). A dendrimer was obtained through a copper-catalyzed azide–alkylidene reaction where two dendrons—either with an azide or an acetylene group—were coupled through the formation of a triazole. The obtained Janus dendrimers displayed a tuning of their self-assembly properties by dispersion in aqueous solution and were targeted for applications such as the transport of gases or ^19^F NMR tracking in MRI (Magnetic Resonance Imaging).

The examples mentioned above provide only a glimpse into the extensive literature available on Janus dendrimers. These structures have been extensively explored, resulting in diverse compositions. For comprehensive reviews of the synthesis and properties of Janus dendrimers, refer to the work by Caminade et al. [14] and Căta et al. [25].

Although known, Janus dendrimers incorporating phosphorus atoms at all branching points remain relatively unexplored, but hold enormous potential in various fields. This comprehensive review aims to present different synthetic approaches to produce phosphorus-based Janus dendrimers, focusing on various coupling strategies employed to construct the desired di-block structures, as well as the targeted applications for these compounds. In addition, the main characterization technique for this type of dendrimer, ^31^P NMR, will be highlighted for the different syntheses.

## 2. Phosphorus Dendrimers Built via Vinyl/Amine Coupling

**Scheme 5:**
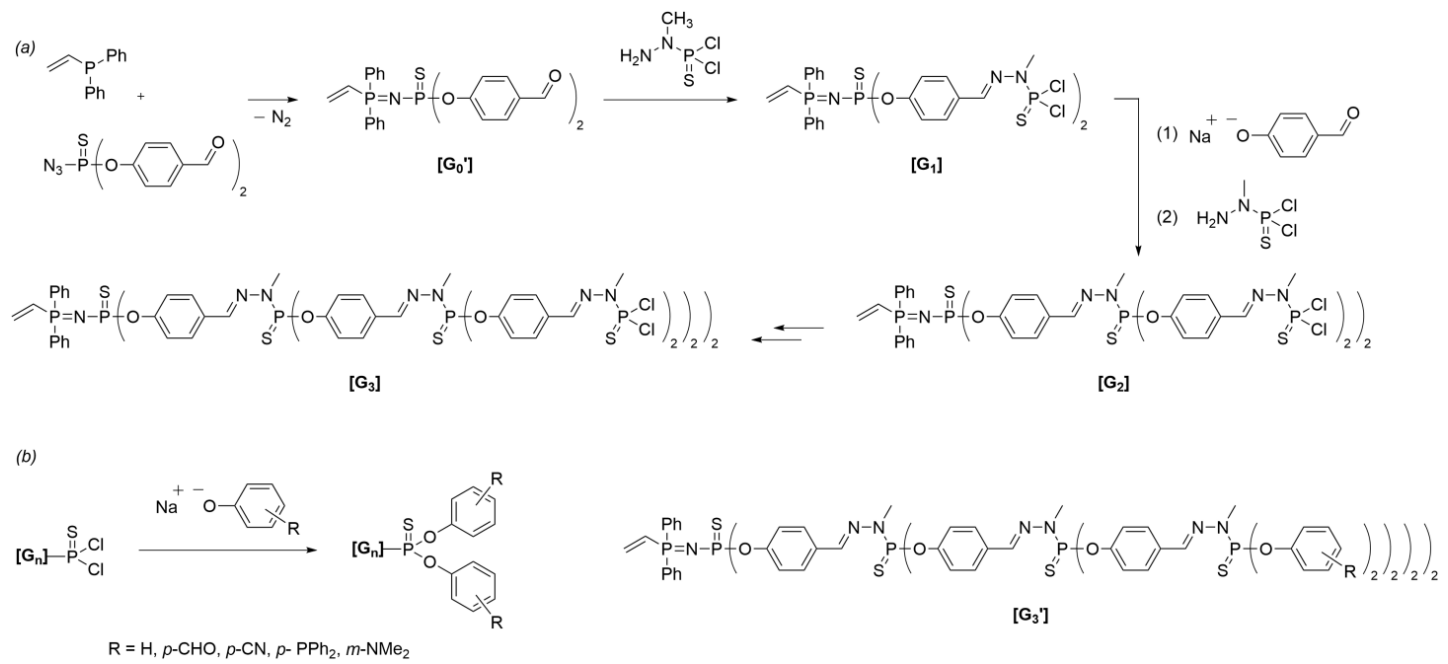
(**a**) Synthesis of polyphosphorhydrazone dendrons bearing a vinyl group at the core up to the third generation. (**b**) Schematic representation of the surface functionalization of PPH dendrons, introducing aldehyde, nitrile, phosphine, or amino derivatives.

**Scheme 6:**
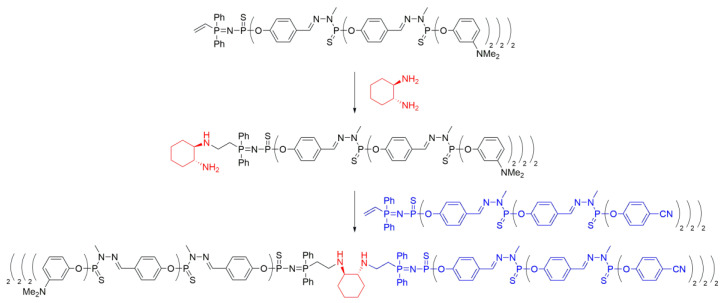
Synthesis of polyphosphorhydrazone Janus dendrimers with a (1*R*,2*R*)-cyclohexane-1,2-diamine core, based on method *ii*.

The pioneering work of the group of Caminade and Majoral presented the first Janus dendrimer incorporating phosphorus atoms at each branching point. The dendrimer was synthesized by combining two dendrons, each possessing a complementary functionality at the core level. Initially, polyphosphorhydrazone (PPH) dendrons were synthesized via a divergent process, featuring a vinyl group at the core and various functional groups on the surface (Scheme 5a) [26]. The vinyl groups were subsequently converted to amine-terminated dendrons through a reaction with a diamine, allowing for subsequent coupling with another dendron. This synthetic approach helped the formation of Janus dendrimers by linking two dendrons together via vinyl/amine coupling, as illustrated in Scheme 6.

In 2000, Maraval et al. [27] reported the synthesis of multidendritic systems based on the Michael addition coupling of amines to dendrons, featuring a vinyl core. The Janus dendrimers were created using an AB_2_-type monomer derived from thiophosphoryl chloride through a two-step process involving the introduction of two benzaldehyde moieties and an azide group. The reaction between the azide terminus and diphenylvinyldiphosphine yielded the desired vinyl core dendron with aldehyde functionalities on the surface. The growth of dendrons followed established methodologies for phosphorus dendrimer synthesis [28,29], involving a condensation reaction between the aldehyde groups and H_2_NN(Me)P(S)Cl_2_. Subsequent growth steps led to the dendrons’ formation, up to the third generation (Scheme 5a). The functionalization of the surface with various substituted phenolic salts resulted in fully characterized dendrons decorated with nitrile groups, phosphines, or amines (Scheme 5b). Dendrons were characterized at each synthetic step using ^1^H NMR, ^31^P NMR, ^13^C NMR, and IR techniques.

Further advancements in Janus dendrimer synthesis involved coupling two dendrons with vinyl groups using the aforementioned diamine method. The vinyl moiety was reacted with an excess of the corresponding diamine (ethylenediamine or (1*R*,2*R*)-cyclohexane-1,2-diamine) to obtain mono-addition products, which were subsequently reacted with a second dendron to yield the desired di-block dendrimers (Scheme 6). Evidence of successful core coupling was observed through an increased symmetry of the core, as confirmed by ^13^C NMR spectroscopy. This methodology allowed for the synthesis of various Janus dendrimers containing different functional groups (nitriles, phosphines, or amines) on their surfaces by combining two types of functional groups.

In 2006, Maraval et al. [30] employed the same vinyl/amine methodology to synthesize dendrimers with multi-charged surfaces. Specifically, they described the synthesis of polycationic, neutral, and polyanionic dendrons by incorporating ammonium tags, carboxylic acid groups, and carboxylates on the surface of the dendrons, respectively. The synthetic approach to obtain dendrons was previously described (refer to Scheme 5). The —P(S)Cl_2_ terminus of the vinyl dendrons was modified with the desired functionalities to generate polyionic systems, which could serve as promising candidates for biological applications [31,32] or layer-by-layer deposition (*see later*).

**Scheme 7:**
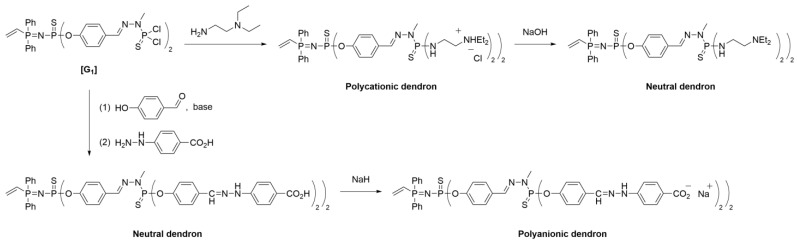
Synthesis of polycationic, neutral, and polyanionic dendrons bearing a vinyl group at the core.

The synthesis of polycationic dendrons (Scheme 7) involved the reaction of the dendritic moieties with *N*,*N*-diethylethylenediamine through a substitution reaction to yield a tetraammonium-functionalized dendron. Hydrogen chloride generated during the process was quantitatively captured by the terminal amines. The progression of the reaction and the purity of the compounds were evaluated using ^31^P NMR, as the coupling with diethylethylenediamine resulted in a significant shift of the terminal phosphorus atom signal from δ = 63.4 ppm to δ = 73.4 ppm. It is important to emphasize the use of stoichiometric quantities of diethylethylenediamine to prevent Michael addition at the vinyl core. Subsequent treatment with NaOH produced the corresponding tetraamine-terminated dendrons. This strategy was employed for higher-generation dendrons, leading to ammonium- and amino-tagged dendrons up to the third generation.

Polyanionic dendrons were synthesized through a three-step surface functionalization process (Scheme 7). The —P(S)Cl_2_ surface of the dendron was reacted with 4-hydroxybenzaldehyde under basic conditions, followed by a reaction with hydrazine benzoic acid to yield dendrons with carboxylic acid termini. Lastly, treatment with NaH resulted in the formation of carboxylate dendrons. The progression of the reactions was monitored using NMR techniques. The formation of the hydrazine derivative was confirmed by ^1^H NMR and IR, as indicated by the disappearance of aldehyde signals and the appearance of a new carboxylic acid peak. ^31^P NMR was used to confirm the integrity of the structure.

**Scheme 8:**
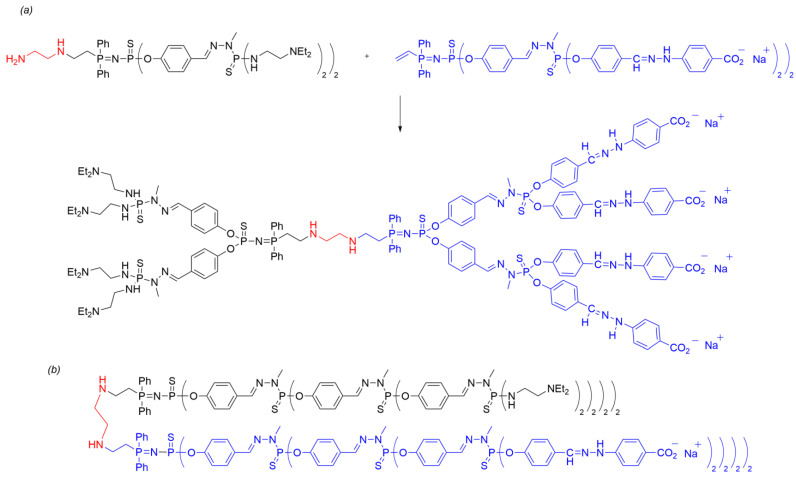
(**a**) Synthesis of a polyanionic Janus dendrimer through amine/vinyl core–core coupling of two second-generation dendrons using method *ii*. (**b**) Linear representation of the third-generation Janus dendrimer with the same structural pattern.

After synthesizing polyanionic, polycationic, and neutral dendrons, Janus dendrimers were prepared by coupling two dendritic moieties through their respective cores. The amine-terminated dendron was reacted with ethylenediamine, undergoing a Michael addition on the vinyl group at the dendron’s core to introduce the desired functionalized core. Subsequently, the coupling of two dendrons possessing vinyl and amino groups at the core was achieved in a biphasic THF/water system by simply combining the two dendrons at 70 °C (Scheme 8a). The progress of the reaction was monitored using ^31^P NMR, as the reaction of the vinyl group linked to the P=N—P=S moiety induced notable shifts in the NMR signals. In particular, the ^31^P NMR chemical shift for the P=N group moves from 14.7 ppm (d, ^2^J_pp_ = 28.0 Hz) in the dendron to 21.7 ppm (d, ^2^J_pp_ = 30.9 Hz) in the Janus dendrimer. This methodology enabled the synthesis of Janus dendrimers up to the third generation (Scheme 8b). In particular, Janus dendrimers of the hybrid generation were also obtained by combining dendrons of different generations [30].

**Scheme 9:**
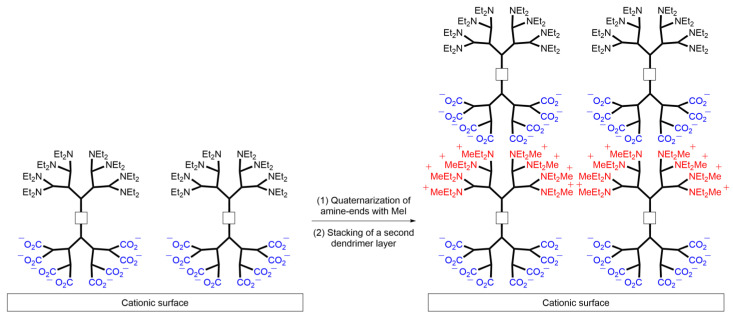
Representation of the interactions between polyionic Janus dendrimers assembled in multilayer systems for applications in materials science.

Lastly, the authors demonstrated the applicability of the polycationic and polyanionic Janus dendrimers for layer-by-layer deposition. As previously shown by Kim et al. in 2005 [33], polyionic dendrimers can be employed to prepare nanotubes and microcapsules by alternately depositing layers of polyanionic and polycationic dendrimers. Preliminary results indicated the successful deposition of a polyanionic Janus dendrimer onto a silica surface pre-coated with a monolayer of 3-aminopropyl dimethylethoxy silane. Similar to the dendrimer depicted in Scheme 9, the deposition of anionic dendrimers onto a positively charged surface occurred through electrostatic interactions, resulting in a neutral surface. The amine terminus was subsequently quaternarized with methyl iodide as an alkylating agent to generate a positively charged surface, which was then covered with the polyanionic dendron part of the Janus dendrimer to assemble the second layer. By repeating this methodology, a thin film was constructed based on the quaternization and adsorption sequences, allowing for the deposition of up to four layers and the creation of nano-objects with potential applications in surface modification.

The spectroscopic and microscopic characterization of these systems was conducted by Lee et al. [34]. UV–visible spectroscopy and atomic force microscopy (AFM) analyses confirmed the uniform formation of films over the entire substrate area, with a consistent increase in film thickness.

## 3. Synthesis of Dendrimers via Staudinger Reaction

The Staudinger reaction is exceptionally versatile in the realm of synthetic chemistry, primarily employed for the synthesis of iminophosphoranes through the reaction between an azide and a phosphine/phosphite [35]. Notably, these reactions typically occur under ambient conditions with simple reaction parameters, leading to high conversions and negligible by-products other than nitrogen gas. These unique characteristics have rendered the Staudinger reaction an outstanding methodology for the synthesis of intricate molecular structures [36,37,38].

**Scheme 10:**
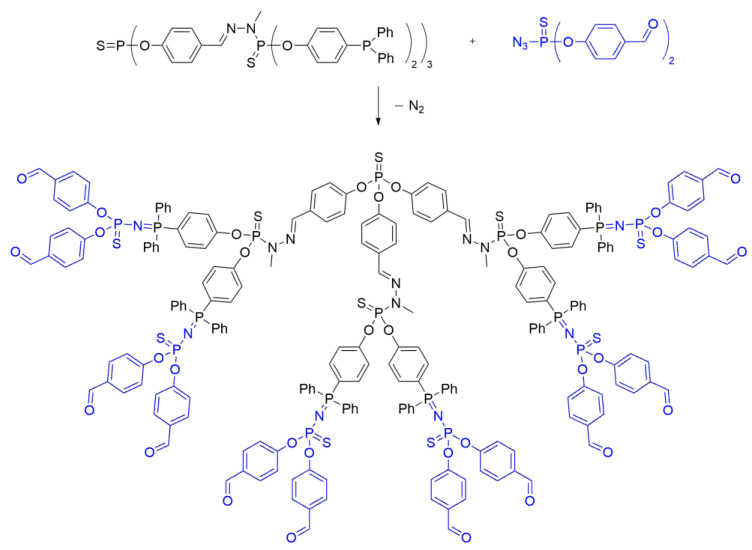
Synthesis of a second-generation polyphosphorhydrazone (PPH) dendrimer by functionalization of the branches through Staudinger reaction.

In the context of dendrimer synthesis, the Staudinger reaction has found application as well. In 2001, Brauge et al. [39] reported the divergent synthesis of a phosphorus dendrimer, incorporating an iminophosphine in its branches. In this approach, an aldehyde-terminated monomer was reacted with a phosphorhydrazide dendron to yield a first-generation dendrimer through condensation reactions, resulting in a dendrimer possessing six terminal diphenylphosphine groups. Subsequently, phosphine groups were reacted with a functionalized azide using the Staudinger reaction, leading to the formation of the second-generation dendrimer (Scheme 10).

**Scheme 11:**
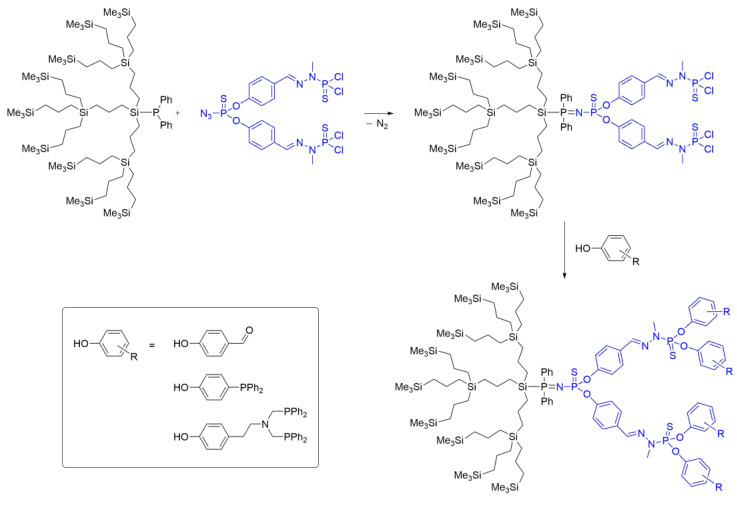
Synthesis of a hybrid carbosilane/PPH Janus dendrimer (based on method *i*) via Staudinger reaction, and the functionality of its surface to accommodate aldehyde and phosphine groups.

Gottis et al. [40] employed a similar strategy for the coupling of dendrons to produce Janus dendrimers. They reported the synthesis of a hybrid carbosilane/PPH dendrimer through the formation of an iminophosphine bond. One dendron featured a thiophosphoryl azide group at the core, terminated with—P(S)Cl_2_ groups, while the other dendron possessed a phosphine moiety at the core and SiMe_3_ terminal groups. These two dendritic units were successfully coupled at room temperature, yielding the desired Janus dendrimer (Scheme 11). The reaction progress was monitored using ^31^P{^1^H} NMR, which revealed the formation of the iminophosphine bond and the generation of a P=N–P=S system through the appearance of distinct signals at δ = 51.9 ppm and δ = 19.8 ppm, corresponding to P=S and P=N, respectively. Janus dendrimers were synthesized up to the second generation using this method.

The resulting Janus dendrimers were subsequently functionalized through the presence of —P(S)Cl_2_ groups on their surfaces. By subjecting the dendrimers to basic conditions, they were functionalized with 4-hydroxybenzaldehyde moieties, allowing for the reactivity of the system to be assessed. The substitution of the —P(S)Cl_2_ groups was monitored using NMR spectroscopy, which indicated a downfield shift from δ = 63.4 ppm (for unsubstituted —P(S)Cl_2_) to δ = 70.0 ppm upon the monosubstitution of the phosphorus atom with the phenol of interest to a —P(S)Cl(OR) system. This substitution pattern ultimately led to the disappearance of the signal at δ = 70.0 ppm, which was replaced by a singlet at δ = 60.7 ppm, corresponding to the fully substituted species. The same methodology was applied to incorporate other ligands, such as tyramine functionalized with two methylene bisdiphenylphosphines, demonstrating a consistent substitution pattern (Scheme 11). These novel phosphine-terminated carbosilane/PPH dendrimers were synthesized with the intention of their potential applications in catalysis in supercritical CO_2_, owing to the coordinating ability of the phosphine ligands present on the Janus dendrimer’s surface.

**Scheme 12:**
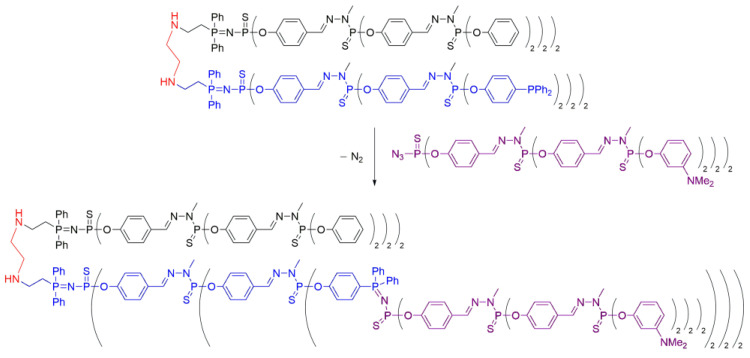
Synthesis of a multiblock Janus dendrimer, with a dendron anchored at the surface of the Janus dendrimer via “click” Staudinger reaction, based on method *iii*.

Interestingly, Maraval et al. [27] described the synthesis of a novel multiblock dendrimer by anchoring a dendron via Staudinger reaction to a previously obtained Janus dendrimer. It was synthesized using the methodology described in Scheme 6. Notably, a Janus dendrimer bearing terminal phosphine groups on one side was utilized for the synthesis of the mentioned multiblock system. As shown in Scheme 12, an azido dendrimer, which served as a building block for the preparation of the Janus dendrimers, was incorporated into the phosphine branches of the dendrimer, forming a P=N—P=S linkage. This demonstrated the versatility of the novel phosphine-ended Janus dendrimers in terms of their derivatization opportunities, yielding to the obtention of highly structural complex systems.

**Scheme 13:**
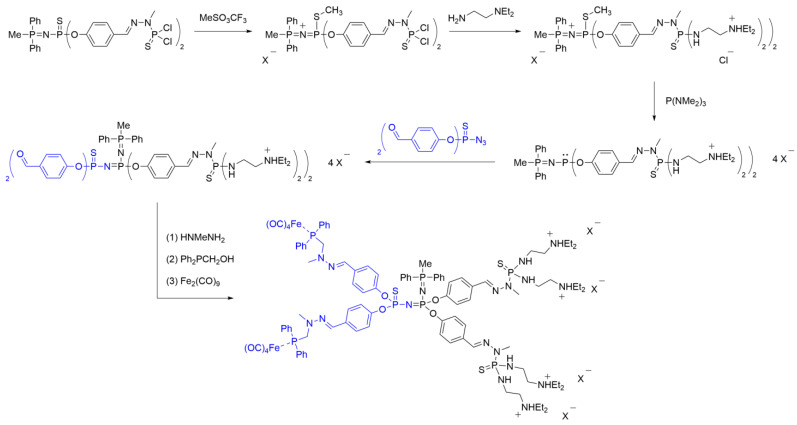
Synthesis of a PPH Janus dendrimer via a novel desulfurization methodology and Staudinger reaction, following method *iii*.

In 2001, a novel methodology for the synthesis of core–core-built Janus dendrimers through Staudinger reaction was reported. The process involved alkylation and the subsequent desulfurization of a dendron P=N—P=S core, followed by a Staudinger reaction with a second desired dendron [41]. The initial dendron was constructed based on an AB_2_ core comprising two benzaldehyde groups and an azide [42]. These functional groups were later on modified through the Staudinger reaction with H_2_NN(Me)P(S)Cl_2_ and the desired phosphine, resulting in the formation of a first-generation dendritic moiety (Scheme 13).

The first-generation dendron was then employed in a novel approach to generate Janus dendrimers through a desulfurization reaction of the —P=S group at the core. As depicted in Scheme 13, the dendron was subjected to the alkylation of the sulfur atom at the core using methyl triflate. The success of this crucial step was confirmed by ^31^P NMR spectroscopy, which revealed the presence of two doublets at δ = 23.8 ppm and δ = 24.1 ppm (^2^J_PP_ = 18.2 Hz), corresponding to the P=N—P—S—Me linkage, as well as a singlet at δ = 62.8 ppm for the terminal —P(S)Cl_2_ group. Subsequently, ionic exchange with NaBPh_4_ and a functionalization of the —P(S)Cl_2_ group with *N*,*N*-diethylethylenediamine were conducted to obtain a polycationic dendron, enabling solubility in a range of solvents, including water. The cationic dendron was further reacted with P(NMe_2_)_3_ to achieve desulfurization. This reaction was confirmed by ^31^P NMR, which displayed two doublets at δ = 144.2 ppm and δ = 10.3 ppm (^2^J_PP_ = 31.6 Hz), corresponding to the core signals.

The activated desulfurized core served as the platform for coupling a second dendron through a Staudinger reaction with a monomer containing two benzaldehyde groups and a reactive azide. This coupling led to the formation of a novel P=N—P=N—P=S core linkage. The resulting Janus dendrimer was utilized for reaction with a di-iron nonacarbonyl compound. To achieve this, the aldehyde groups were reacted with excess methylhydrazine, followed by treatment with Ph_2_PCH_2_OH, yielding a dendrimer with terminal phosphine groups. Potentially, the prepared dendrimer containing phosphine groups at the surface could be used for the complexation of metals, such as palladium, for their potential applications in catalysis.

Finally, the desired Janus dendrimer with ammonium tags and incorporating iron complexes (Fe(CO)_4_) attached to a phosphine was obtained (Scheme 13). The formation of the Fe@dendrimer was confirmed by a significant deshielding of the phosphine signal, shifting from δ = -22.9 ppm to δ = 67.5 ppm [38].

## 4. Synthesis via Coupling of AB_5_ Cyclotriphosphazene Systems

AB_5_ compounds derived from the reactivity of hexachlorocyclotriphosphazene possess one function different from the five others. Such types of compounds have garnered significant attention in the scientific literature due to their facile syntheses [43]. The synthetic approach for generating these dendrons involves the asymmetric functionalization of the core [44], resulting in type compounds denoted as “A” and “B.” The “A” group serves as a linkage point for coupling with another dendron, while the “B” group represents the functional terminal groups. The synthetic methodologies employed to achieve the desired AB_5_ systems are diverse, and stem from two distinct approaches based on the substitution of the N_3_P_3_Cl_6_ core: either five P—Cl bonds are reacted with one reagent and then the last one with a second reagent, or vice versa, by first reacting one Cl with one reagent and the remaining five with a second reagent. The asymmetry of these compounds has led to the development of a great range of dendrons and dendritic structures, yielding to a plethora of compounds with different applications [45]. For a comprehensive review of the syntheses and applications of AB_5_ derivatives in the creation of phosphorus dendrons within the fields of biology, material science, catalysis, and sensors, refer to Zibarov et al. [45].

**Scheme 14:**
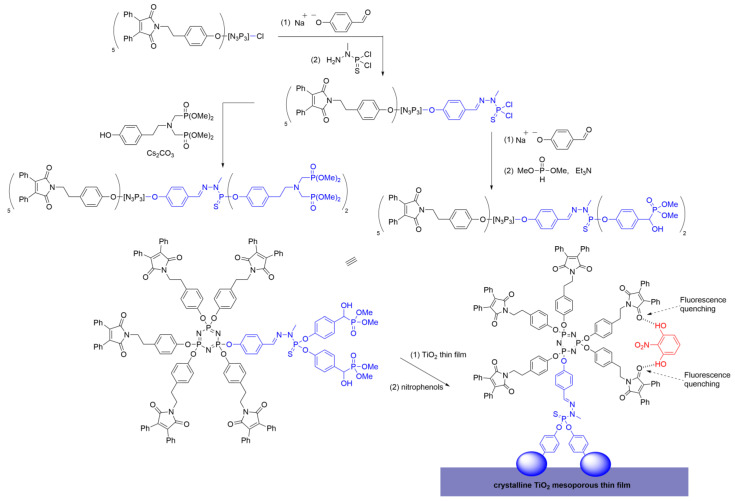
Functionalization of a polyphosphorhydrazone Janus dendrimer with different phosphonates, and grafting to a TiO_2_ thin film, used as a fluorescent sensor for nitrophenols.

An intriguing example is the work described by Martinez-Ferrero et al. [46], wherein dendrimers were synthesized as hybrid optical sensors grafted onto mesoporous titania nanocomposites. An AB_5_ dendron was synthesized using N_3_P_3_Cl_6_ in a two-step process: first, the core was reacted with one equivalent of 4-hydroxybenzaldehyde to achieve the monosubstitution of one P—Cl bond, and second, the remaining five P—Cl bonds were reacted with maleimide-based phenolic chromophores. The successful substitution was confirmed through NMR and IR spectroscopy. The ^1^H and ^13^C NMR spectra exhibited multiple signals due to the asymmetric arrangement of the dendron branches around the core. The AB_5_ core was subsequently functionalized with H_2_NN(Me)P(S)Cl_2_ to enable the further modification of the dendritic structure. The terminal —P(S)Cl_2_ group was decorated with various phosphonate groups, known for their stability in forming complexes with metal oxides (Scheme 14).

Considering the Janus dendrimers prepared, novel hybrid materials were created by combining the fluorescent properties of the maleimide derivative and anchoring the material onto a nanocrystalline mesoporous titania thin film. The primary attribute of these novel materials is their applicability as fluorescent probes. Simultaneously, titania films serve as excellent supports for grafting organic molecules due to their chemical composition, and their mesoporous cavities and optical properties do not interfere with those of the dendrimer. The designed systems exhibit a significant quenching efficiency toward phenolic OH groups, thus suggesting their potential application as detectors for hazardous phenolic compounds, as illustrated in Scheme 14.

**Scheme 15:**
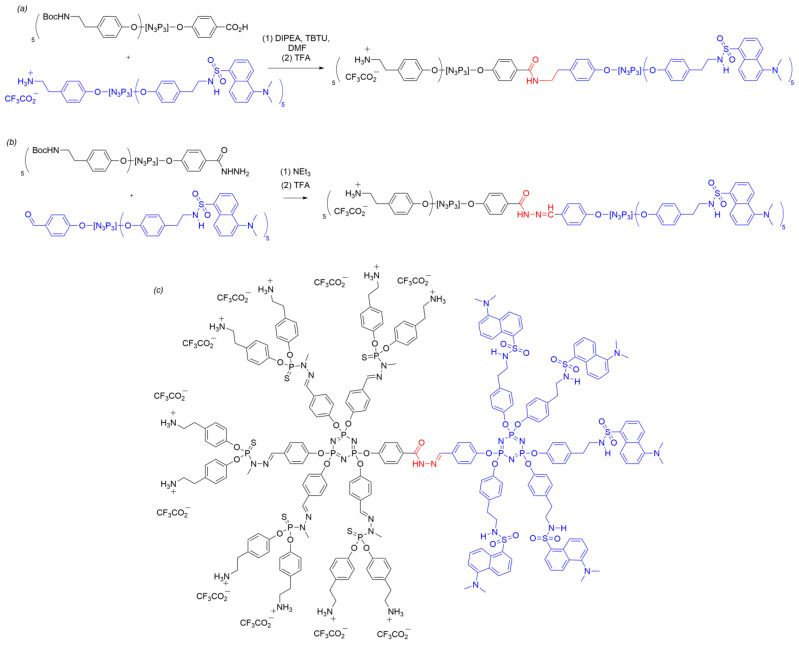
Synthesis of Janus dendrimers (based on method *i*) bearing dansyl groups in one end and ammonium tags in the other through an (**a**) amide coupling; or (**b**) hydrazone linker; and (**c**) an expanded structure of a hybrid second-generation/first-generation Janus dendrimer with a hydrazone linker.

Despite extensive research on the synthesis of dendrons using the AB_5_ derivative of cyclotriphosphazene, there are only two instances of employing these dendrons in the synthesis of Janus dendrimers, as described by Fuchs et al. in 2015 [47]. Utilizing the aforementioned methodology, two dendrons were synthesized, one containing five amino groups protected with *tert*-butyloxycarbonyl (Boc) groups and one carboxylic acid function, while the other contained five dansyl derivatives and one primary ammonium group (Scheme 15). Dansyl groups were chosen as the functional moiety for fluorescent labeling, as previously reported in the literature for fluorescent probes in dendrimers [48,49,50]. The synthesis of the Janus dendrimer proceeded through amide formation. Firstly, the carboxylic acid group of the first dendron was activated using TBTU (*O*-(benzotriazol-1-yl)-1,1,3,3-tetramethyluronium tetrafluoroborate) in the presence of DIPEA (*N*,*N’*-diisopropylethylamine). Subsequently, the activated dendron was reacted with the ammonium salt of the second dendron under basic conditions to form an amide bond (Scheme 15a). Finally, the resulting Janus dendrimer was treated with an excess of trifluoroacetic acid in dichloromethane to remove the Boc protecting group, resulting in a dendrimer with dansyl groups on one side and ammonium tags on the other.

Another strategy to synthesize Janus dendrimers with dansyl and ammonium tags involved the use of a hydrazone bond (Scheme 15b). This approach was chosen because of the anticipated superior stability of hydrazones compared to that of amines in condensation reactions. For this purpose, two new dendrons were prepared: the first containing five Boc-protected primary amines and a hydrazide group, and the second containing five dansyl groups and one aldehyde group. The coupling of the two AB_5_ dendrons was achieved under basic conditions, resulting in the desired Janus dendrimer connected by a hydrazone linker. The obtained Janus dendrimer was again treated with trifluoroacetic acid to remove the Boc protecting group. The progress of the reaction was monitored by NMR and IR spectroscopy, observing the disappearance of the aldehyde signal and the formation of the hydrazone bond.

Both synthesized Janus dendrimers were designed for applications in the fields of material science [51] and biology [52,53,54], leveraging the presence of fluorescent dansyl groups and ammonium tags, which provide water solubility. The fluorescent properties of the dendrimers were evaluated in dioxane as the solvent, demonstrating similar absorption and emission maxima wavelengths with a substantial Stokes shift resulting from the presence of the dansyl groups. A minimal spectral overlap was observed between the absorption and emission spectra, a highly advantageous feature for future biological applications, as it enables an improved separation of the probe’s inherent light and the light scattered by the sample.

Of note, in 2011, Furer et al. conducted a density functional theory (DFT) study, as well as the IR and Raman spectra on the structure, of a dendron featuring five dansyl groups and a carbamate [55]. Computational studies involving structural optimization and normal mode analysis were performed, showing good agreement between the calculated geometrical parameters, harmonic vibrational frequencies, and experimental data. The calculations revealed that the dendron possesses a concave lens structure with a slightly non-planar cyclotriphosphazene core. Additionally, the intensity of the most prominent bands in the IR spectrum was reproduced by the calculations. Overall, the combined experimental and computational studies provided a detailed understanding of the dendron structure for the first time.

## 5. Conclusions

Phosphorus Janus dendrimers offer a promising alternative to conventional dendritic systems due to their highly versatile functional groups The incorporation of multiple functionalities within a single dendrimer enables their utilization in multifaceted applications. In contrast to traditional organic Janus dendrimers, phosphorus analogs possess a notable advantage in terms of facile characterization, as illustrated in this review. Indeed, ^31^P NMR is a very precious tool for assessing the purity of phosphorus-containing dendritic structures [56]. The characterization of dendritic compounds is never trivial, due to their repetitive structure, which often hampers any NMR characterization since the chemical environment is strictly identical at all layers. However, a slight difference in the density at each layer may slightly modify the geometry around the branching elements. ^1^H and ^13^C NMR are not sensitive to these subtle differences, contrarily to ^31^P NMR, which is able to afford a different (sharp) signal for each dendritic layer, at least up to five different layers. Furthermore, the P=N-P=S linkages shown in several Schemes in this review are characterized by a set of two doublets, for which both the chemical shifts and the coupling constant are very sensitive to their environment, and in particular to the reactions that occur on the constituents of this linkage, but also on the reactions that occur on the substituents of this linkage. Such behavior is particularly useful to characterize the sophisticated dendritic structures that are the Janus dendrimers.

The pivotal aspect in the advancement of phosphorus Janus dendrimers lies in the coupling of dendrons, primarily accomplished through a core-to-core assembly, using straightforward and efficient reactions. Since the synthesized macromolecules from their corresponding dendrons can be challenging, it is highly desirable to employ quantitative reactions that do not generate side products. In this comprehensive review, we have presented various methodologies described so far for the preparation of these systems. The most extensively explored approach involves the coupling of dendrons possessing vinyl and amine groups through a Michael addition reaction. However, alternative reactions have also been investigated, such as the formation of amide or hydrazone bonds, as well as the utilization of the Staudinger reaction.

Despite the considerable progress made in the development of these systems, there remains ample scope for synthesizing such architectures using novel approaches. In this regard, the integration of a “click” chemistry between azide and alkyne holds tremendous potential, as it could enable the generation of complex dendritic structures using straightforward methodologies.

Lastly, the phosphorus Janus dendrimers discussed in this review have demonstrated applicability in various fields, particularly in materials science. Nonetheless, numerous other applications could be targeted in the future, particularly in critical areas such as catalysis.

## Data Availability

Not Applicable.
